# Treatment of Diabetic Macular Edema with Intravitreal Antivascular Endothelial Growth Factor and Prompt versus Deferred Focal Laser during Long-Term Follow-Up and Identification of Prognostic Retinal Markers

**DOI:** 10.1155/2018/3082560

**Published:** 2018-10-01

**Authors:** Birgit Weingessel, Kata Miháltz, Andreas Gleiss, Florian Sulzbacher, Christopher Schütze, Pia V. Vécsei-Marlovits

**Affiliations:** ^1^Department of Ophthalmology, Hietzing Hospital, Wolkersbergenstrasse 1, Vienna, Austria; ^2^Karl Landsteiner Institute for Process Optimization and Quality Management in Cataract Surgery, Wolkersbergenstrasse 1, Vienna, Austria; ^3^Center for Medical Statistics, Informatics and Intelligent Systems, Medical University of Vienna, Vienna, Austria

## Abstract

**Purpose:**

Long-term follow-up of patients with diabetic macular edema (DME) treated with intravitreal antivascular endothelial growth factor (anti-VEGF) combined focal laser and identification of prognostic morphological characteristics.

**Methods:**

Prospective clinical trial (50 treatment-naive eyes) with DME randomized 1 : 1 receiving intravitreal ranibizumab (0.5 mg/0.05 ml) and prompt grid laser compared with ranibizumab and deferred laser. Morphological characteristics potentially relevant for prognosis were assessed at baseline, month 6, month 9, and years 1, 2, 3, 4, and 5 of follow-up.

**Results:**

Although functional results were slightly higher in the prompt group at week 12 (0.5; 20/40 Snellen (SD = 0.04, 0.3 logMAR) versus 0.4; 20/50 Snellen (SD = 0.04, logMAR: 0.4), *p*=0.4) and month 9 (prompt group: 0.5; 20/40 Snellen (SD = 0.03, 0.3 logMAR) versus deferred group: 0.4; 20/50 Snellen (SD = 0.04, 0.4 logMAR), *p*=0.4), these were statistically insignificant. There was no significant benefit regarding functionality during long-term follow-up in the prompt group compared to the deferred group. BCVA in the eyes with clusters of hyperreflective foci in the central macular region was inferior compared with the eyes without these alterations at year 5 (0.39; 20/50 Snellen, (SD = 0.25, 0.4 logMAR) versus 0.63; 20/80 Snellen (SD = 0.22, 0.2 logMAR), *p* < 0.01).

**Conclusion:**

Grid laser and ranibizumab therapy are effective in DME management during the long-term follow-up. Intraretinal hyperreflective material in SD-OCT is negatively related to BCVA.

## 1. Introduction

Diabetic macular edema (DME) is the most common cause of visual impairment in diabetic patients [1]. More than four hundred million adults are affected by diabetes mellitus worldwide. Ninety percent of diabetic patients will have some form of retinopathy 25 years following diagnosis [2].

It is estimated that 25% of individuals with type 1 diabetes and 15% of patients with type 2 diabetes develop macular edema and that one half of these patients lose two or more lines of visual acuity (VA) during the disease course [2, 3].

Elevated levels of vascular endothelial growth factor (VEGF) have shown to promote permeability of retinal vasculature leading to macular edema in diabetic patients [4, 5].

Besides regulation of blood glucose level and blood pressure, DME has been treated with focal/grid laser until recently that reduced the risk of moderate vision loss (defined as a loss of more than 3 lines of VA) by 50% in 3 years. In patients with central macular edema with baseline VA of <0.5, 11% of the eyes gained more than 3 lines in one year and 16% more than 3 lines following 3 years [4–6].

With the introduction of anti-VEGF agents, a milestone in the management of DME has been achieved by lowering intraocular VEGF levels and thereby reducing macular edema resulting in restoration of visual function. In the Intravitreal Aflibercept for Diabetic Macular Edema (VIVID/VISTA) studies, randomized diabetic patients received aflibercept or focal/grid laser treatment. Mean VA improvement was lowest in the laser groups (+0.9 and +0.7) compared with that of the aflibercept arms for VIVID and VISTA, respectively [7].

The Ranibizumab for Diabetic Macular Edema (RISE/RIDE) studies showed that laser monotherapy was not as effective in improving VA compared with ranibizumab treatment that was effective in rapidly and sustainably improving VA and in reducing the risk of further vision loss and in improving macular edema in patients with DME [8].

The Ranibizumab Plus Prompt Or Deferred Laser Or Triamcinolone Plus Prompt Laser For Diabetic Macular Edema (DRCR.net Protocol I Study) indicated that nonetheless ranibizumab plus deferred laser reached best VA results; it is significant to note that ranibizumab plus prompt laser-treated eyes reached a similar 5-year VA with lower number of injections needed (median: 17 versus. 13, resp.) and a median of 3 focal/grid photocoagulation managements. Furthermore, the original laser group achieved best outcomes regarding central macular thickness (CMT) reduction [9].

Therefore, it has been shown that macular laser therapy may still play an important role as an adjuvant therapy because it is able to improve macular thickness outcomes and to reduce the number of intravitreal injections needed. However, the determination of morphological retinal features that are relevant for functional prognosis seems indispensable when aiming for the identification of more individualized treatment procedures for DME patients.

The study presented here provides additional information to functional/anatomical outcomes in eyes with DME treated with anti-VEGF combined with prompt compared to deferred laser therapy and identifies prognostically relevant morphological characteristics using high-resolution spectral domain optical coherence tomography (SD-OCT) during long-term follow-up.

## 2. Materials and Methods

This prospective longitudinal randomized interventional clinical trial included 50 treatment-naive patients (50 eyes) with diabetic macular edema (DME). Patients were recruited at the Department of Ophthalmology, Hietzing Hospital, Vienna, Austria. Signed informed consent was obtained from each patient, and the character of the study was explained in detail preceding patient inclusion. The local ethics committee approved the study protocol that followed the ethical tenets of the Declaration of Helsinki.

### 2.1. Inclusion and Exclusion Criteria

Patients underwent a comprehensive ophthalmological examination that included slit lamp biomicroscopy, funduscopy, tonometry (intraocular pressure-IOP), fluorescein angiography (FA), and SD-OCT (Spectralis HRA + OCT, Heidelberg Engineering, Heidelberg, Germany). In case of DME that was detected clinically and on FA as well as in SD-OCT (diffuse macular edema with central retinal thickness (CRT) ≥300 *µ*m involving the center of the macular area), patients were included into the present study following informed consent. Inclusion criteria were further best-corrected visual acuity (BCVA) between 0.06; 6/120 (1.2 logMAR) and 0.63; 20/32 (0.20 logMAR). Data were analyzed every 6 weeks until month 6, followed by month 9 and years 1, 2, 3, 4, and 5.

Investigators who measured visual acuity were all masked to the respective study arm. The method used for measuring visual acuity was Snellen charts.

The eyes with other retinal diseases (i.e., age-related macular degeneration (AMD) and associated choroidal neovascularization (CNV), cystoid macular edema (CME) of other origin (e.g., uveitis, Irvine–Gass syndrome, and retinal vein occlusion), or retinal dystrophies) were excluded from the study. Further exclusion criteria were previous treatment of DME with laser and/or intravitreal injections with anti-VEGF or triamcinolone or panretinal photocoagulation at least 3 months prior to the baseline visit. Moreover, patients who had intraocular surgery at least 6 months prior to the first visit were excluded from the study. Myocardial infarction or stroke 6 months or less prior to baseline as well as hypertension >180/110 mmHg and unstable blood glucose levels (HbA1C ≥9.0%) were exclusion criteria. An upper limit of CRT of ≥600 *µ*m in SD-OCT was defined for study inclusion in order to eliminate presence of eyes with extensive structural damage.

### 2.2. Randomization and Treatment Methods

The eyes of eligible patients were randomized into 2 groups (ratio 1 : 1), and they either received ranibizumab and focal/grid laser on the same day (“prompt” group, 25 eyes, 50%) or ranibizumab one week prior to laser treatment (“deferred” group, 25 eyes, 50%). Randomization was performed with the Randomizer® software available at the Medical University of Vienna (http://www.meduniwien.ac.at/randomizer/).

When both the eyes of a patient were affected by diabetic retinopathy (DR) and met the inclusion criteria, the left eye was randomized to prompt laser treatment and anti-VEGF, and the right eye received deferred laser therapy in addition to intravitreal anti-VEGF.

Ranibizumab (0.5 mg/0.05 ml) was injected via pars plana using a 30-gauge needle following topical application of betadine solution and anesthesia with oxybuprocaine. Retreatment was performed on the same day of the clinical examination if retreatment criteria were met. These followed the DRCR.net protocol [10] that continued retreatment every 6 weeks until no further functional and morphological improvement was reached, which was defined as a decrease in CRT of <10% and an increase in BCVA of <5 letters compared with the last intravitreal anti-VEGF injection.

Focal laser treatment was performed using argon laser photocoagulation (VISULAS 532s®, Carl Zeiss Meditec) operating at 532 nm. Laser therapy was accomplished only once and not repeated during the observation period. Light was focused on the retina applying a Mainster Focal/Grid Laser contact lens (Ocular Instruments, Bellevue, WA, USA). Focal photocoagulation was used for treatment of macular edema as described by ETDRS [5]. No spots were applied closer to the foveal avascular zone than 500 *μ*m. Duration used was 100 ms, and the treatment goal was to obtain burns as light as possible that were only barely visible at the level of the outer retina. Direct treatment of microaneurysms was performed. In case of development of proliferative DR (neovascularization on the optic disc (NVD) or elsewhere (NVE)) detected clinically or on FA, panretinal photocoagulation was initiated.

All patients who were primarily included into this study are and will be continuously followed and treated as needed at the Department of Ophthalmology, Hietzing Hospital, Vienna, Austria.

### 2.3. Imaging Procedures and Data Analyses

High-resolution retinal imaging that acquires three-dimensional cross-sectional retinal datasets was performed using Spectralis OCT. The device obtains a variable quantity of B-scans and implements an eye tracker to minimize artifacts that are caused by eye motion. The system combines infrared (IR) fundus autofluorescence (FAF), FA, and a scanning laser ophthalmoscopy (SLO) unit. In the present study, macular raster scans measuring 6000 *μ*m in diameter were used. Macular volume scans were generated automatically using the software provided by Spectralis (version 6.3.2.0).

Accumulations of clusters of hyperreflective material in the central macular B-scans of an SD-OCT dataset (1 cluster = accumulation of a minimum of 3 adjacent hyperreflective foci) were analyzed and compared by 2 independent readers regarding frequency of occurrence in both treatment groups. Presence of intraretinal exudative foci was correlated to the functional outcome during follow-up.

### 2.4. Statistical Analyses

CRT and BCVA were determined as primary outcome measures at designated time points. BCVA is specified in Snellen and logMAR units and standard deviation (SD) is given as Snellen equivalents. Prognostic relevant morphological parameters were identified qualitatively and quantitatively (%). BCVA is given in decimal, Snellen, and logMAR units.

Sample size calculation was based on a Wilcoxon rank-sum test (nQuery-Version 6.01). For comparison analyses between BCVA, CRT, and frequency of intraretinal hyperreflective foci among treatment groups and between different points in time, the paired *t*-test and Mann–Whitney *U* test were used. Risk of retreatment was investigated in a logistic regression model. Excel 2014, MedCalc (version 13.3.3.0), and Past Project (version 3.06) were used to perform statistical analyses. Statistical significance was defined as *p* < 0.05.

## 3. Results

The mean age of patients was 66.2 (SD = 9.0 years, min = 49 years, and max = 84 years). There was no significant difference regarding age between treatment groups. Twenty patients were female, and 30 were male.  Table 1 summarizes baseline characteristics of patients of each treatment group.

### 3.1. Functional and Morphological Parameters

**Figure 1:**
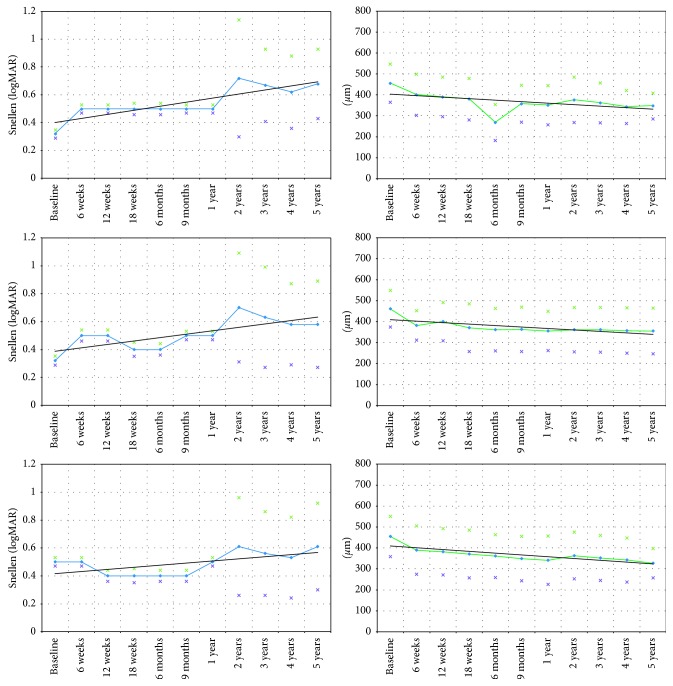
Best-corrected visual acuity (BCVA) and central retinal thickness (CRT) in eyes with diabetic macular edema (DME) treated with intravitreal antivascular endothelial growth factor (anti-VEGF) combined with prompt or deferred focal macular laser. Although functional results in eyes treated with immediate anti-VEGF therapy combined with laser at week 12 (prompt treatment group: 0.5; 20/40 Snellen (SD = 0.04, 0.3 logMAR) versus deferred treatment group: 0.4; 20/50 Snellen (SD = 0.04, logMAR: 0.4), *p*=0.4) and month 9 (prompt group: 0.5; 20/40 Snellen (SD = 0.03, 0.3 logMAR) versus deferred group: 0.4; 20/50 Snellen (SD = 0.04, 0.4 logMAR), *p*=0.4)) were slightly better; no meaningful functional benefit was observed in the prompt group during long-term follow-up. BCVA = best-corrected visual acuity. CRT = central retinal thickness. Black lines represent trend lines. Stars represent standard deviation. *p* values indicate change compared with baseline. (a) BCVA-entire study population (50 eyes). (b) CRT-entire study population (50 eyes). (c) BCVA-prompt/deferred (light blue line) group (25/25 eyes). (d) CRT-prompt/deferred (light green line) group (25/25 eyes).

Figure 1 and Table 2 summarize results regarding BCVA and CRT outcomes. Overall, mean BCVA changed from 0.32; 20/63 Snellen (SD = 0.03, 0.5 logMAR) at baseline to 0.68; 20/32 Snellen (SD = 0.25, 0.2 logMAR) at year 5 of follow-up (*p* > 0.1). The mean BCVA changed from 0.32; 20/63 Snellen (SD = 0.03, 0.5 logMAR) at baseline to 0.58; 20/40 Snellen (SD = 0.31, 0.2 logMAR) at year 5 in the prompt group (*p* < 0.001) and from 0.32; 20/63 Snellen (SD = 0.03, 0.5 logMAR) at baseline to 0.61; 20/32 Snellen (SD = 0.31, 0.2 logMAR) at year 5 in the deferred treatment group (*p* < 0.001). Difference of BCVA between treatment groups was statistically insignificant at year 5 (*p*=0.3). Although our results revealed slightly better functional results in the beginning of the treatment phase in the prompt group at week 12 (prompt group: 0.5; 20/40 Snellen (SD = 0.04, 0.3 logMAR) versus deferred group: 0.4; 20/50 Snellen (SD = 0.04, logMAR: 0.4), *p*=0.4) and month 9 (prompt group: 0.5; 20/40 Snellen (SD = 0.03, 0.3 logMAR): versus deferred group: 0.4; 20/50 Snellen (SD = 0.04, 0.4 logMAR), *p*=0.4) of follow-up, differences in visual acuity between both groups were insignificant during the long-term follow-up. There was no significant difference in CRT when comparing the prompt and deferred treatment group at week 12 (*p*=0.08) and month 9 (*p*=0.3) of follow-up. Average CRT values of the entire study population changed from 457.2 *µ*m (SD = 90.7 *µ*m) at baseline to 348.3 *µ*m (SD = 62.0 *µ*m) at the end of the observation period (*p* < 0.01). Mean CRT decreased from 460.3 *µ*m (SD = 87.4 *µ*m) at baseline to 354.4 *µ*m (SD = 108.9 *µ*m) in the prompt group (*p* < 0.001) and from 454.1 *µ*m (SD = 95.6 *µ*m) at baseline to 327.6 *µ*m (SD = 69.7 *µ*m, *p* < 0.001) in the deferred group at year 5.

**Figure 2:**
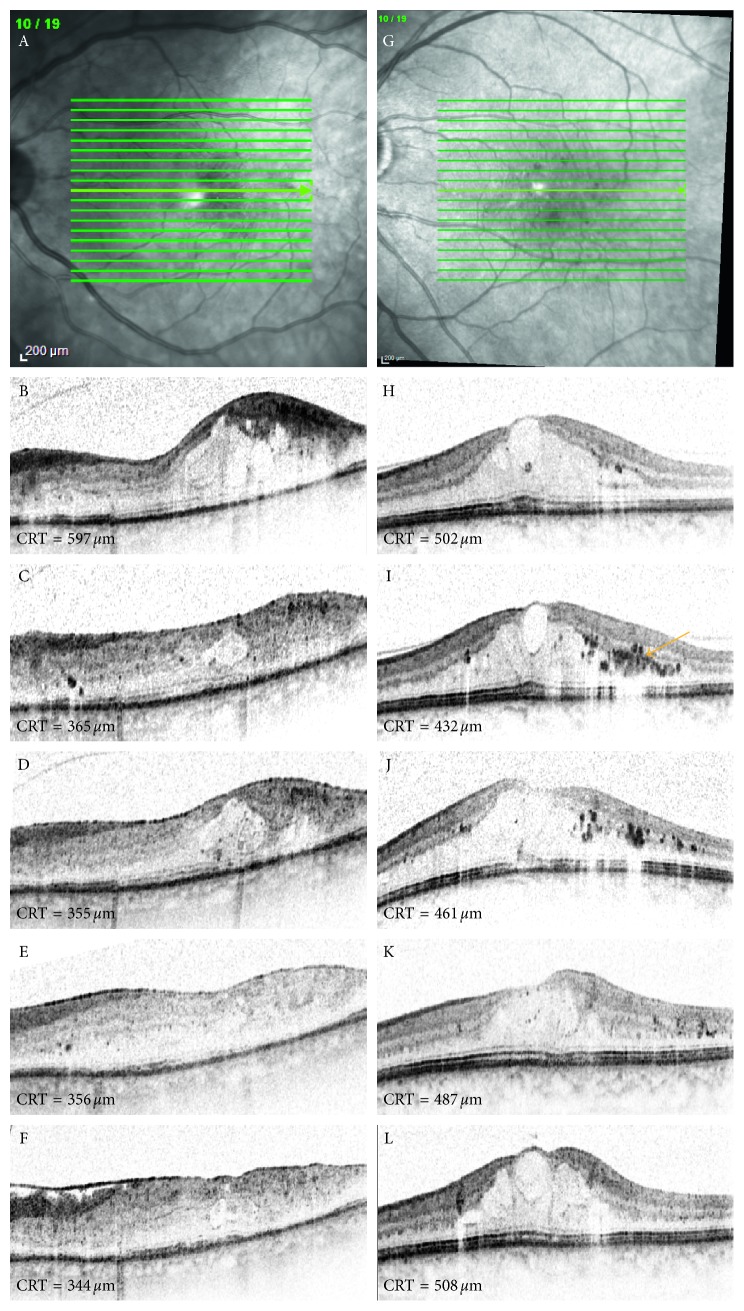
Spectral domain optical coherence tomography (SD-OCT) images of the left eye of 2 patients treated with ranibizumab and prompt focal laser therapy (A–F) or ranibizumab in addition to deferred laser (H–L) at baseline (B, H), 6 months (C, I), 1 year (D, J), 2 years (E, K), and 5 years (F, L) following baseline. SD-OCT images indicate a more rapid and consistent decrease of central retinal thickness (CRT) values in the eye treated with prompt laser therapy compared with the eye randomized for deferred focal laser treatment. In this example, the decrease in CRT is particularly evident in the eye that received prompt laser therapy in the beginning of the treatment phase (until month 6) compared with the eye that was scheduled for the deferred group. Note that the baseline CRT was similar in both eyes of the different treatment arms. The arrow indicates clusters of hyperreflective intraretinal material.

In general, reduction of retinal edema was more distinct in the eyes of the prompt group (particularly at an early disease stage) as compared with the deferred group (Figure 2).

Until month 6 of follow-up (prompt treatment group: total of 49 intravitreal injections needed, potential maximum cumulative number of injections = 119; deferred treatment group: 47 injections needed, potential maximum cumulative number of injections = 111), there was a statistically insignificant likelihood of a lower retreatment rate in the deferred group compared with the prompt group (odds ratio: 0.43, 95% confidence interval (CI): 0.11–1.65), though statistically insignificant (*p*=0.21).

Baseline BCVA (*p*=0.19) and CRT (*p*=0.75) values did not differ significantly between the prompt or deferred treatment groups.

No severe ocular adverse events (i.e., endophthalmitis or persistent arterial nonperfusion) occurred in this study.

**Figure 3:**
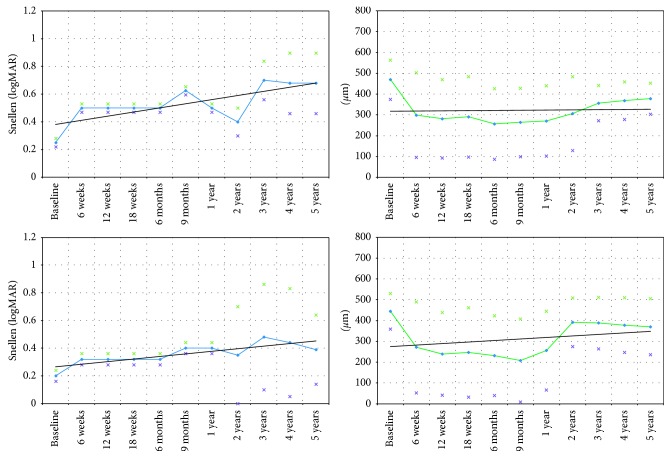
Best-corrected visual acuity (BCVA) and central retinal thickness (CRT) in eyes with diabetic macular edema (DME) treated with intravitreal antivascular endothelial growth factor (anti-VEGF) and focal macular laser. Data represent eyes revealing clusters of intraretinal hyperreflective material as detected on spectral domain optical coherence tomography (SD-OCT) and eyes without these morphological changes. BCVA in eyes with clusters of hyperreflective foci in the central macular region detected by SD-OCT was significantly inferior compared with eyes without intraretinal hyperreflective foci at year 5 of follow-up (0.39; 20/50 Snellen (SD = 0.25, 0.4 logMAR) versus 0.63; 20/32 Snellen (SD = 0.22, 0.2 logMAR), *p* < 0.01). BCVA = best-corrected visual acuity. CRT = central retinal thickness. Black lines represent trend lines. Stars indicate standard deviation. *p* values indicate change compared with baseline. (a) BCVA in eyes with (22 eyes) and without (20 eyes, light blue line) intraretinal hypherreflective foci in SD-OCT. (b) CRT in eyes with (22 eyes) and without (28 eyes, light green line) intraretinal hypherreflective foci in SD-OCT.

Regarding clusters of intraretinal material detected in the central macular area by OCT imaging, these hyperreflective foci consistently correlated with lipid exudates seen on biomicroscopy. BCVA in the eyes with clusters of intraretinal foci in the central macular area (one cluster depicts an accumulation of a minimum of 3 adjacent hyperreflective foci as evident in SD-OCT B-scans (22 eyes, 44%, Figure 2)) was inferior compared with the eyes without (28 eyes) clusters of intraretinal hyperreflective material within retinal layers prior to treatment (0.20; 20/100 Snellen (SD = 0.03, 0.7 logMAR) versus 0.25; 20/80 Snellen (SD = 0.03, 0.6 logMAR), *p*=0.25, Table 3). At 5 years of follow-up, this development of inferiority of visual function was maintained (0.39; 20/50 Snellen (SD = 0.25, 0.5 logMAR) versus 0.68; 20/32 Snellen (SD = 0.22, 0.2 logMAR), *p*=0.25, Figure 3, Table 3). Frequency and intensity of hyperreflective foci in outer retinal layers decreased until year 5 of follow-up compared with baseline.

## 4. Discussion

Diabetic retinopathy (DR) is a retinal vascular disorder that occurs as a complication of diabetes mellitus (DM) and is the leading cause of blindness in the developed world [1, 10, 11]. The condition results in retinal ischemia (i.e., microaneurysms, hemorrhages, cotton wool spots, intraretinal microvascular abnormalities, or macular edema) and/or signs of increased retinal vascular permeability. Loss of vision is a consequence of various pathophysiological mechanisms including neovascularization that may cause vitreous hemorrhage, retinal detachment, or capillary nonperfusion. Retinopathy occurs in most patients with DM of longer duration, though its incidence can be reduced by aggressive control of hyperglycemia and hypertension [12].

The recent advent of antivascular endothelial growth factor (anti-VEGF) has revolutionized the management of DR with a significant improvement in the overall prognosis. In the management of diabetic macular edema (DME) intravitreally administered ranibizumab has been shown to achieve favorable treatment outcomes in various randomized prospective clinical trials, reaching significant functional and morphological improvements [9, 13]. However, the development towards more individualized treatment strategies in eyes with DME demands the identification of prognostically relevant morphological features that allow for a better understanding regarding treatment response during the long-term follow-up.

Hence, it was the aim of the current study to evaluate functional and morphological outcomes in the eyes with DME treated with ranibizumab and immediate focal laser therapy compared with eyes treated with intravitreal ranibizumab and deferred laser. This study showed that treatment with anti-VEGF in addition to focal laser is effective and safe in the management of DME during a long-term period of 5 years. Results revealed that the use of prompt laser in addition to intravitreal ranibizumab is not advantageous compared with anti-VEGF therapy during the long-term follow-up. However, as our findings revealed that functional results in the prompt group were slightly higher (though not statistically significant) compared with the deferred treatment group in an earlier treatment phase; this suggests that focal laser therapy in the eyes with DME is justified.

Previous findings [14–16] are in accordance with the results of our study, signifying consistency of the treatment regimen.

The analysis of Protocol I data presented by Gonzales et al. [14] determined whether early visual acuity (VA) response to ranibizumab in diabetic macular edema is associated with long-term outcome and showed that ranibizumab ± laser therapy resulted in similar rates (∼40%) of BCVA improvement following 12 weeks of treatment. The eyes with suboptimal early BCVA response showed poorer long-term visual outcomes than eyes with pronounced early response [14].

Similar to the READ-2 study (Nguyen et al. [13]), the Ranibizumab Monotherapy or Combined with Laser versus Laser Monotherapy for Diabetic Macular Edema (RESTORE) study achieved favorable functional and morphological results using intravitreal ranibizumab combined with laser [15].

Furthermore, the effectivity of intravitreal anti-VEGF combined with laser has been shown in the Diabetic Retinopathy Clinical Research Network trial at the 1-year of follow-up. At 3 years, prompt laser was not better compared with deferring laser for 24 weeks or more [16].

The study presented here further showed that morphological features like center-involving intraretinal hyperreflective material detected by SD-OCT may represent a negative predictive factor in the eyes with DME because the initially observed inferior VA of the eyes revealing intraretinal hyperreflective foci at baseline remained until the end of the follow-up period (0.39; 20/50 Snellen (SD = 0.25) versus 0.68; 20/63 Snellen (SD = 0.22), *p*=25, Table 3). Hyperreflective intraretinal spots define exudates as visible clinically and were described previously by Vujosevic et al. [17]. Hyperreflective structures seen in SD-OCT correlated to hard exudates observed in biomicroscopy in our study. The eyes that displayed hyperreflective structures in outer retinal layers in the present study showed a marked decrease of these alterations following treatment with anti-VEGF during follow-up. As BCVA generally improved as treatment with ranibizumab progressed, it may be suggested that a reduction of intraretinal hyperreflective foci followed by anti-VEGF therapy may at least partly represent a positive predictive morphological feature in the eyes with DME. This assumption has also been suggested previously [18], it and is supported by our findings during an observation period of 5 years.

Intraretinal hyperreflective foci were further less frequently present in the prompt treatment group compared with the deferred group as treatment progressed (57% versus 43% of cases with clusters of intraretinal hyperreflective structures at the end of the observation period), potentially signifying a therapeutic benefit as compared with deferred laser. However; this finding needs to be confirmed in larger prospective clinical trials.

Described hyperreflective intraretinal alterations may further be clinically relevant in an early stage of DR as it has been described previously that these hyperreflective spots are present in diabetic eyes even when retinopathy is undetectable clinically, increasing in number when DR progresses. This hypothesis is supported by the inferior VA in eyes with hyperreflective intraretinal alterations in our study. In addition, Niu et al. [18] showed that the area and amount of hyperreflective spots can serve as a potential discriminant indicator for the severity of DR. The current study confirmed this hypothesis in a long-term clinical setting. Our findings regarding a clinically predictive value of hyperreflective intraretinal foci are further supported by Kang et al. [19] who demonstrated that the number of hyperreflective foci in outer retinal layers visible in SD-OCT at baseline might predict the final VA in DME.

Although it has been shown by Kang et al. [19] that higher total and low-density lipoprotein cholesterol levels were associated with presence of hyperreflective foci in SD-OCT, the precise origin of these hyperreflective foci remains to be shown in histopathological studies or by using tissue-specific retinal imaging modalities (i.e., polarization sensitive OCT (PS-OCT)) [20] that provide inherent tissue contrast visualization. Intraretinal exudative material may also contain intraretinal macrophages and represent the level of inflammatory activity in DME. This may explain the negative correlation between intraretinal hyperreflective foci and visual function.

It remains to be shown if the eyes with extensive exudative material in the central macular region and reduced BCVA in our study would profit from an individualized and more intense anti-VEGF treatment strategy, as has been suggested by others previously [21].

Limitations of the current study are the relatively low number of patients and the lack of identification of other morphological parameters in OCT (i.e., integrity of the retinal pigment epithelium (RPE) or the identification and segmentation of individual outer retinal layers in SD-OCT) that may serve as relevant prognostic markers in eyes with DME and will be addressed in future investigations. Furthermore, treating physicians were not masked according to the group of patients, which is considered as a study limitation.

Another drawback of this study is that patients with DME in addition to neovascularization on the disc (NVD) or elsewhere (NVE) were not analyzed separately or compared to the study population presented here. Regarding treatment with anti-VEGF in DME, only ranibizumab was assessed in our investigation; though evaluation of intraretinal morphological changes following treatment with bevacizumab, aflibercept, dexamethasone, or triamcinolone should be considered in future investigations (including the identification of functionally relevant morphological characteristics) in order to possibly implement individualized patient management strategies during a long-term follow-up period.

Although our study showed no severe ocular adverse events like endophthalmitis or arterial nonperfusion, enhanced investigation regarding ophthalmological or systemic adverse events would further have increased the validity of our study.

The lack of a comparison analysis between the eyes treated with anti-VEGF alone and the eyes receiving anti-VEGF in addition to focal laser is another drawback of the current study.

An automated segmentation analysis software algorithm used for quantification of intraretinal hyperreflective foci in all B-scans of dense volume SD-OCT datasets in this study would further have been advantageous.

It may be suggested that change in BCVA primarily resulted due to treatment with focal laser and anti-VEGF, as only 6 eyes (12%) of the current study received cataract surgery during the observation period.

To conclude, the study presented here showed that combined treatment with ranibizumab and focal laser effectively reduces visual loss due to DME, which is a major sight-threatening cause in diabetic patients. Our results regarding functional long-term development are in conjunction with those of several previous clinical trials [7–9,14–16] that evaluate the efficacy of anti-VEGF and laser treatment in DR, favoring anti-VEGF. Nevertheless, macular laser can still be applied and seems particularly favorable in the beginning of the treatment phase. Furthermore, the combined therapy with anti-VEGF and macular laser can exploit the synergistic effects of both therapies, leading to a simpler and more practical management of patients during the long-term follow-up. Our results suggest that anti-VEGF treatment in addition to prompt laser is favorable because of sparing an additional visit for patients. Moreover, the findings of our study may aid in the identification of prognostic morphological features that are clinically relevant for patients with DME and potentially significant for the development of prospective individualized therapeutic procedures in order to reduce the burden of frequent clinical management for affected patients.

## 5. Conclusions

The present study showed that grid laser and ranibizumab treatment are effective in DME management during the long-term follow-up. Overall, there was no meaningful functional benefit in the long run when comparing prompt versus deferred focal laser combined with anti-VEGF in the long run. Morphological characteristics like intraretinal hyperreflective material shown in SD-OCT are negatively related to functionality in DME patients.

## Figures and Tables

**Table 1:** Baseline parameters of patients with diabetic macular edema (DME) enrolled in the current study.

*Prompt laser group*	
Age (mean/SD)	65.3/8.3
Gender (male/female)	17/11
Visual acuity (Snellen/SD/logMAR)	0.32/0.03/0.50
Central retinal thickness (*µ*m)	460.3/67.3
*Deferred laser group*	
Age (mean/SD)	67.2/9.7
Gender (male/female)	18/9
Visual acuity (Snellen/SD/logMAR)	0.32/0.03/0.50
Central retinal thickness (*µ*m)	454.1/95.6

SD = standard deviation.

**Table 2:** Functional ((best-corrected visual acuity (BCVA)) and morphological (central retinal thickness (CRT)) results in eyes with diabetic macular edema (DME, 50 eyes, top) treated with intravitreal ranibizumab and immediate focal laser therapy (prompt group, 25 eyes, middle) and eyes treated with intravitreal ranibizumab and deferred laser (25 eyes, bottom).

	Baseline	6 weeks	12 weeks	18 weeks	6 months	9 months	1 year	2 years	3 years	4 years	5 years
*Entire study population*											
BCVA	0.32	0,50	0.50	0.50	0.50	0.50	0.50	0.72	0.67	0.62	0.68
SD	0.03	0,03	0.03	0.04	0.04	0.03	0.03	0.42	0.26	0.26	0.25
logMAR (Snellen)	0.50 (20/40)	0.30 (20/63)	0.30 (20/63)	0.30 (20/63)	0.30 (20/63)	0.30 (20/63)	0.30 (20/63)	0.20 (20/100)	0.25 (20/80)	0.30 (20/63)	0.20 (20/100)
CRT	457.20	401.6	391.3	381.10	269.60	358.90	351.90	377.40	363.00	343.80	348.30
SD	90.70	98.40	94.20	99.30	85.70	88.30	93.00	108.20	95.20	78.70	62.00

*Prompt treatment group*											
BCVA	0.32	0.50	0.50	0.40	0.04	0.50	0.50	0.70	0.63	0.58	0.58
SD	0.03	0.04	0.04	0.05	0.04	0.03	0.03	0.39	0.36	0.29	0.31
logMAR (Snellen)	0.50 (20/40)	0.30 (20/63)	0.30 (20/63)	0.40 (20/50)	0.40 (20/50)	0.30 (20/63)	0.30 (20/63)	0.20 (20/100)	0.20 (20/100)	0.30 (20/63)	0.30 (20/63)
CRT	460.30	381.60	399.40	370.20	360.70	362.80	354.70	360.70	360.70	357.00	354.40
SD	87.40	70.20	91.50	113.50	101.50	106.10	94.10	105.80	106.90	108.00	108.90

*Deferred treatment group*											
BCVA	0.32	0.50	0.40	0.40	0.40	0.40	0.50	0.61	0.56	0.53	0.61
SD	0.03	0.03	0.04	0.05	0.04	0.04	0.03	0.35	0.30	0.29	0.31
logMAR (Snellen)	0.50 (20/40)	0.30 (20/63)	0.40 (20/50)	0.40 (20/50)	0.40 (20/50)	0.40 (20/50)	0.30 (20/63)	0.30 (20/63)	0.30 (20/63)	0.30 (20/63)	0.30 (20/63)
CRT	454.10	389.70	382.00	370.20	360.70	348.80	340.90	363.40	352.40	342.10	327.60
SD	95.60	114.80	110.10	113.50	101.50	106.10	114.80	111.10	107.30	104.50	69.70

*Note*. BCVA = best-corrected visual acuity, SD = standard deviation, and CRT = central retinal thickness.

**Table 3:** Functional (best-corrected visual acuity (BCVA)) and morphological (central retinal thickness (CRT)) results in eyes with diabetic macular edema (DME) following treatment with intravitreal ranibizumab and focal laser therapy that reveal clusters of hyperreflective foci in the central macular area (27 eyes, 54%) and eyes without hyperreflective intraretinal foci (23 eyes, 46%). Results show that BCVA in eyes revealing clusters of hyperreflective foci in the central macular region detected by spectral domain optical coherence tomography (SD-OCT) was significantly inferior compared with eyes without clusters of hyperreflective intraretinal foci at year 5 (0.39 Snellen (SD = 0.25, 0.4 logMAR) versus 0.63 Snellen (SD = 0.22, 0.2 logMAR), *p* < 0.01)

	Baseline	6 weeks	12 weeks	18 weeks	6 months	9 months	1 year	2 years	3 years	4 years	5 years
*Eyes revealing clusters of hyperreflective foci in the central macular area in SD-OCT*											
BCVA	0.32	0.50	0.50	0.50	0.50	0.50	0.50	0.72	0.67	0.62	0.68
SD	0.03	0.03	0.03	0.04	0.04	0.03	0.03	0.42	0.26	0.26	0.25
logMAR (Snellen)	0.50 (20/40)	0.30 (20/63)	0.30 (20/63)	0.30 (20/63)	0.30 (20/63)	0.30 (20/63)	0.30 (20/63)	0.20 (20/100)	0.20 (20/100)	0.20 (20/100)	0.20 (20/100)
CRT	457.20	401.60	391.30	381.10	269.60	358.90	351.90	377.40	363.00	343.80	348.30
SD	90.70	98.40	94.20	99.30	85.70	88.30	93.00	108.20	95.20	78.70	62.00
*Eyes without clusters of hyperreflective foci in the central macular area in SD-OCT*											
BCVA	0.32	0.50	0.50	0.40	0.40	0.50	0.50	0.70	0.63	0.58	0.58
SD	0.03	0.04	0.04	0.05	0.04	0.03	0.03	0.39	0.36	0.29	0.31
logMAR (Snellen)	0.50 (20/40)	0.30 (20/63)	0.30 (20/63)	0.40 (20/50)	0.40 (20/50)	0.30 (20/63)	0.30 (20/63)	0.20 (20/100)	0.20 (20/100)	0.30 (20/63)	0.30 (20/63)
CRT	460.30	381.60	399.40	370.20	360.70	362.80	354.70	360.70	360.70	357.00	354.40
SD	87.40	70.20	91.50	113.50	101.50	106.10	94.10	105.80	106.90	108.00	108.90

*Note*. BCVA = best-corrected visual acuity, SD = standard deviation, and CRT = central retinal thickness.

## Data Availability

Data of the current study were acquired and processed as described in the methods section of the manuscript.
